# Study of volatile compounds in Greek pistachio (*Pistacia vera* L. ‘Aegina’ cultivar) oils using Soxhlet and ultrasound assisted extraction

**DOI:** 10.1016/j.heliyon.2023.e15623

**Published:** 2023-04-20

**Authors:** Lydia Valasi, Evangelia C. Zafeiri, Ioanna Thanou, Christos S. Pappas

**Affiliations:** Laboratory of Chemistry, Department of Food Science & Human Nutrition, Agricultural University of Athens, Iera Odos 75, 11855, Greece

**Keywords:** *Pistacia vera*, Volatile, Extraction, Soxhlet, UAE, Temperature

## Abstract

Headspace solid-phase microextraction followed by gas chromatography-mass spectrometry (HS-SPME/GC-MS) represents the most used solvent-free methodology for the characterization of the complex and heterogeneous mix of volatile compounds. The present study investigates the differences in volatile profile of pistachio oils ‘Aegina’ cultivar extracted with two different techniques, ultrasound assisted extraction (UAE) and Soxhlet. Differences were observed both in the pistachio oil yield and the composition of the volatile compounds among these two groups of samples, which were significantly influenced due to the different thermal conditions. In terms of pistachio oil yield, the Soxhlet extraction technique was proven more efficient (52.5–68.2% w/w) than the UAE type (28.2–42.6% w/w). A total of 34 and 30 volatile compounds were identified for UAE and Soxhlet, respectively. The main ones associated with UAE were α-pinene, octane and decane, while the volatiles formed as a consequence of Soxhlet extraction were decane, nonanal and (*E*)-2-decenal. Terpenes' concentrations were found decreased in Soxhlet's samples, but hydrocarbons and aldehydes were significantly increased in these samples. Numerous studies concluded in common results. However, this article is the first to explore the influence of different extraction types on the volatile profile of the unique flavour and odor pistachio oil ‘Aegina’ cultivar.

## Highlights

• The volatiles of pistachio oil ‘Aegina’ cultivar (two extraction ways) were studied.
• Between the extraction types, ultrasound assisted extraction gave lower yield.
• The different thermal conditions resulted in different volatile compounds.
• Long time processing under high temperature rouse formation of roasted compounds.

## Graphical abstract

**Figure:**
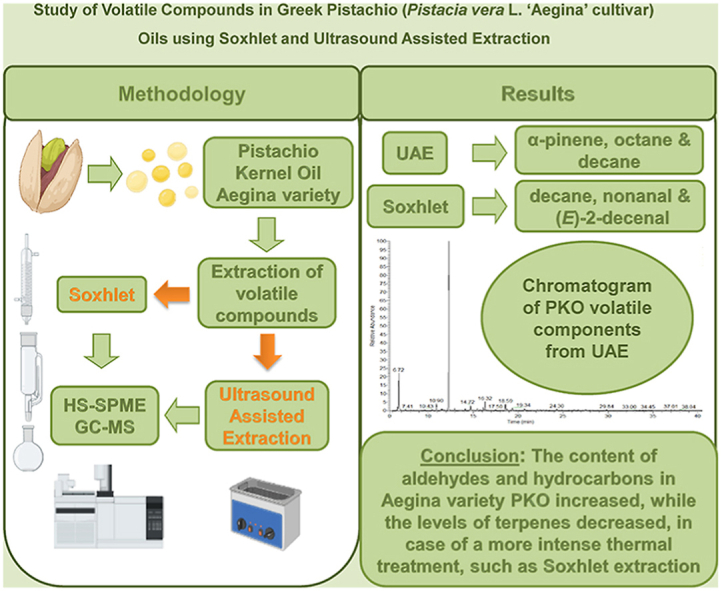

## 1 Introduction

Members of the Pistacia genus belong to the cashew family (Anacardiaceae) and contain at least eleven species from which *Pistacia vera* L. (pistachio) is the only edible commercial species [1].

The pistachio nut is an important agricultural commodity for several countries. Latest data set the United States of America, Iran, Turkey and China on top of the pistachio producers with annual yield quantities of 523,900 tonnes, 135,000 tonnes, 119,355 tonnes and 78,818 tonnes, respectively [2]. Pistachio nut has gained attention due to its special organoleptic characteristics [3] and its high contents of some nutrients and health promoting compounds, such as monounsaturated fatty acids (24.534 g/100 g dried kernels), vitamins (8.75 mg/100 g dried kernels), minerals (1704.37 mg/100 g dried kernels), sterols (89 mg/100 g dried kernels) and polyphenols (185 mg gallic acid equivalents/100 g dried kernels) [[4], [5], [6], [7], [8], [9], [10]].

The pistachio kernel oil (PKO) is not described by the current Committee on Fats and Oils of the Codex Alimentarius, but it is prized as a ‘specialty oil’ owing to its beneficial effects on human health [11]. PKO content ranges from 50 to 60% w/w in kernels, depending mainly on cultivar, crop year, and geographic location [12,13]. Triglycerides constitute the major components of PKO, in which monounsaturated and polyunsaturated fatty acids are present in high levels [14]. The presence of other micronutrients, such as tocopherols (8.4 mg/100 g dried kernels) and plant pigments (18.1 mg/100 g dried kernels), has been also documented [9,15]. Due to PKO's high unsaturation and micronutrient levels, an extreme care needs to be taken to prevent quality degradation reactions during extraction processes [16].

Cold pressing methods of extraction maintain the unique flavour and the special odor of ‘specialty oils’. Mechanical or hydraulic cold pressing is a simple extraction technique that, since heating is not applied to oil seeds during the pressing, can yield very pure, nutritionally rich, and sensorally acceptable oils, which do not require refining and can be consumed directly [16]. Other methods of extraction, such as ultrasound assisted extraction (UAE) and Soxhlet extraction, require the appliance of heating and the use of organic solvents (hexane, petroleum ether, dichloromethane) resulting in higher oil yield. Especially, Soxhlet extraction presents an increased oil yield [17,18]. Long periods of sonication (greater than 40 min) at high energy level (above 20 kHz) could facilitate the generation of unintended changes and free radicals in the extracted compounds [19]. Extraction with supercritical liquids, such as carbon dioxide (CO_2_), is another widely used extraction technique. Although its high cost of equipment is deterrent, its oil yield resembles the high yield levels of Soxhlet [20]. Cold pressing does not affect the raw characteristics of nut oils but lacks in yield as compared with solvent extraction. However, the Soxhlet extraction includes a degradation of pistachio oil due to the implementation of high temperature [17,18].

Qualitative and quantitative differences of volatile compounds may be observed due to the different types of extraction. Volatiles are degraded by the combination of the high temperature and the long extraction time of Soxhlet technique. The UAE technique provides lower operating temperature and shorter extraction time leading to absence of thermal degradation, no energy loss and extraction of oils with high antioxidant bioactivity [21]. The effect of heat treatment was evident according to Rodríguez-Bencomo et al. [22]. Specifically, the single-roasting and double-roasting process on the pistachios resulted in a change of some volatiles’ content, such as an increase in the content of hexanal, d-limonene, *p*-cymen-8-ol, benzaldehyde and a production of new volatile compounds based on pyrazin.

Headspace solid-phase microextraction (HS-SPME) is widely employed to isolate and pre-concentrate volatiles prior to gas chromatography–mass spectrometry (GC-MS) analysis. HS-SPME has several advantages over other sample preparation techniques, including its minimum or zero amount of solvents, ease of automation, and small sample size requirements [23,24]. The appliance of HS-SPME/GC-MS has been proven a useful tool in the analysis of volatile compounds [[25], [26], [27], [28], [29], [30], [31]]. It is not surprising that a large amount of papers are devoted to the use of GC-MS in the comparison of different sample pre-treatment techniques [[32], [33], [34]].

Compared to the conventional Soxhlet extraction, the advanced UAE, as a non-thermal extraction technique, is based on the appliance of shorter extraction times and thus less energy requirement, the efficient performance at lower temperature which simultaneously retains the quality of the extract and the reduction of solvent consumption [35]. Moreover, UAE is considered environmentally friendly and may be scalable [36]. Based on the above mentioned facts and the gap of knowledge concerning volatiles of Greek pistachio oils (‘Aegina’ cultivar), it comes the need to obtain valuable information about the chemical composition of the volatile profile in Greek pistachio oils (‘Aegina’ cultivar) and investigate the compounds' differences using Soxhlet and UAE techniques.

## 2 Material and methods

### 2.1 Samples

A quantity of 45 dehulled Greek pistachio samples (*Pistacia vera* cultivar ‘Aegina’), at an average moisture content of 5–7% w/w, were obtained from several cultivation areas of Greece (2017–2018 harvesting seasons). The shells of pistachios were removed manually and each sample was finely ground using a food processor, sieved (500–800 μm) and stored (−20 °C in vacuum-sealed plastic bags) prior to chemical analysis.

### 2.2 Reagents

Hexane, used as solvent for the extraction of pistachio oil, was purchased from Merck (Darmstadt, Germany). Methyl decanoate, used as internal standard (IS) in GC-MS analysis, was purchased from Sigma-Aldrich (Steinheim, Germany). Kovats retention indices (KRI) values of volatile compounds were calculated using n-alkane (C7–C24) standards (Supelco, Bellefonte, USA). All reagents were of analytical grade purity.

### 2.3 Extraction procedures

#### 2.3.1 Soxhlet extraction

Pistachio oil was extracted in a Soxhlet apparatus (AOAC Official Method 948.22). An amount of 4 g sieved pistachio kernel sample was placed in a cellulose extraction cartridge and positioned in the extraction chamber just over the collecting flask under a reflux condenser. Then, 250 mL hexane were added to the flask and the solvent was heated to produce vapour which condensed under cool running water and dropped back into the cellulose extraction cartridge that held the pulverized sample. The reflux was maintained severally (6 h at 70 °C). Finally, the oil extract was obtained back from the heating flask.

#### 2.3.2 Ultrasound assisted extraction (UAE)

UAE of pistachio oil by hexane was performed using a Grant single frequency ultrasonic bath (Beaver Falls, Pennsylvania, USA) of internal dimensions 266 × 235 × 345 mm and four disc transducers. The ultrasonic frequency was adjusted at 35 kHz. During sonication, 8 g of sieved pistachio kernel sample were mixed with 170 mL of solvent in a conical flask for 15 min at ambient temperature (25±1 °C). Subsequently, the extract was vacuum-filtered through a Buchner funnel. The above extraction and filtration processes were performed twice more, each time keeping the same solid residue and adding 170 mL of hexane at a time. The three filtrates of a sample were collected in the same bottle. For each sample, three repetitions of the above described procedure were implemented.

#### 2.3.3 Determination of lipid content

After the extractions, either Soxhlet or ultrasound assisted, the solvent was evaporated under vacuum (Laborota 400 efficient, Link Lab, Heidolph). The PKO, containing the volatile compounds, was weighed and collected in an amber vial to avoid light exposure until the GC-MS analysis was performed. In each way of extraction and for each repetition, the total lipid content was calculated through the ratio of the weight of the extracted oil to the weight of the sample taken (Equation (1)). The mean value of the pistachio oil percentage and the standard deviation were calculated for each sample from its triplicates. Pistachio oils were stored at −20 °C in the dark, to preserve their organoleptic and chemical characteristics [37,38].

(1) % pistachio oil = [weight of extracted oil (g)/weight of sieved pistachio kernel (g)] x 100

### 2.4 Analysis of pistachio oil volatile compounds

For the isolation of PKO volatile compounds, an efficient method based on HS-SPME/GC-MS was developed. The extraction procedure of volatiles as well as the choice of the SPME fiber coating was according to Ojeda-Amador, Fregapane and Desamparados [39].

#### 2.4.1 HS-SPME conditions

For each sample, 1 g of pistachio oil was placed into a 15 mL screwed glass vial (22.7 × 86 mm) which was sealed with a polytetrafluoroethylene (PTFE)/silicone septum cap. The sealed vial was left to equilibrate in a thermostatic water bath for 5 min at 50 °C. Subsequently, a 50/30 μm divinylbenzene/carboxen/polydimethylsiloxane (DVB/CAR/PDMS) Stable-Flex fiber (Supelco, Bellefonte, PA, USA) was exposed to the equilibrated headspace of the vial for 30 min under constant stirring at 50 °C. The fiber was previously conditioned in the GC injector for 30 min at 260 °C (as recommended by the manufacturer) to remove any volatile contaminants.

#### 2.4.2 GC-MS conditions

After headspace sampling, the fiber was immediately inserted into the gas chromatograph for thermal desorption with an inlet temperature of 250 °C and a 0.8 mm injector liner (SGE International Pty Ltd., Ringwood, Australia) for 3 min. The analysis was performed in split mode; initially with a 1/1 ratio and a 1/20 ratio after 1 min.

A Trace ULTRA gas chromatograph (Thermo Scientific Inc., Waltham, MA, USA) coupled to a mass spectrometer (DSQ II) was used to separate the volatiles. Separation of the compounds was performed on a non-polar Trace TR-5MS column (30 m, 0.25 mm ID, 0.25 μm film thickness, Thermo Fisher Scientific, USA) with a static phase of 5% phenylpolysilphenylene-siloxane. Helium with a flow rate of 1.0 mL/min under constant pressure was used as the carrier gas. The analysis program lasted for 40.50 min. The GC oven temperature started and maintained at 40 °C for 5 min. It was then programmed to increase from 40 °C to 150 °C at a rate of 4 °C/min without remaining in that temperature. It then increased to 260 °C at a rate of 20 °C/min and remained at that temperature for 2.50 min. Regarding the MS transmission line, the ion source and the interface system temperatures were 200 °C and 260 °C, respectively, with a mass range of 35–400 amu and an ionization energy of 70 eV.

For each sample, the volatile compounds' analysis was carried out three times, while a control sample (blank injection) was analyzed before each sample to prevent fiber contamination. For calculation of samples’ KRI, a solution of a series of homologous alkanes (C7–C24) was analyzed under the above GC-MS conditions.

#### 2.4.3 Volatile compounds’ identification

The volatile compounds were identified in the XCalibur software by comparing the mass spectra and the KRI of the samples with: a) those of in-house or commercially available libraries (Wiley 275, NBS 75K, NIST (NIST/EPA/NIH Mass Spectral Library, NIST 05 data version, software version 2.0d), Adams) and b) the KRIs of the homologous alkanes (C7–C24). The perfect match of the experimental with the bibliographic KRI gave a value of R. Match 999. The non-matched peaks resulted in a value of zero. Overall, a value of 900 or higher resulted in a great match, values of 700–900 a good match and less than 600 a very bad match. For each way of extraction and each repetition, the content of each component was determined as a percentage of its area relative to the total area of all the volatile components (%). For each sample and for both extraction types, the mean value of each volatile compound's content and its standard deviation were calculated from the three repetitions.

## 3 Results and discussion

### 3.1 Pistachio oil yield

Table 1 presents the results of the pistachio oil yield based on both Soxhlet and UAE techniques. The mean yield fluctuated between 52.5% and 68.2% w/w (Soxhlet) and between 28.2% and 42.6% w/w (UAE) and the average percentage together with the standard deviation were found to be 60.5 ± 3.0 and 34.3 ± 3.8% w/w, respectively. The lipid content percentage from the Soxhlet technique is in absolute agreement with another study of the same Greek pistachio cultivar and the same type of extraction which ranged between 57.5% and 62.2% w/w [40]. The yield results of the two pistachio oil types of extraction proved that the UAE gave a lower efficiency than the Soxhlet technique (Table 1). T-test for equality of means was performed using the type of extraction as the grouping variable. The significance value (S·V.) was lower than 0.05 (p-value<0.05), so the variable was statistically significant.

**Table 1:** Pistachio oil yield (g PKO/100 g sieved pistachio kernel) of 45 samples based on Soxhlet and UAE techniques.

Samples	Soxhlet^a^	UAE^b^
1	63.5 ± 4.4^c^	33.4 ± 0.6
2	62.6 ± 1.3	31.3 ± 4.2
3	62.5 ± 3.3	39.1 ± 0.2
4	68.2 ± 5.1	42.6 ± 3.4
5	66.7 ± 1.0	39.2 ± 0.4
6	61.2 ± 2.2	32.1 ± 4.5
7	61.5 ± 1.7	38.4 ± 3.6
8	60.1 ± 3.0	40.1 ± 0.8
9	60.3 ± 1.9	31.7 ± 1.1
10	60.2 ± 0.3	40.1 ± 0.1
11	64.1 ± 2.2	33.7 ± 3.4
12	64.8 ± 2.4	38.1 ± 0.3
13	62.5 ± 10.7	39.1 ± 0.5
14	60.0 ± 1.1	33.3 ± 2.9
15	59.7 ± 4.4	31.4 ± 3.7
16	60.9 ± 4.9	30.8 ± 5.4
17	62.2 ± 1.8	37.9 ± 0.6
18	61.9 ± 0.8	38.2 ± 0.3
19	63.2 ± 2.5	37.2 ± 0.6
20	57.5 ± 6.8	35.5 ± 4.3
21	60.0 ± 5.9	31.6 ± 3.3
22	57.7 ± 5.3	38.4 ± 2.5
23	57.4 ± 9.0	30.8 ± 4.3
24	60.4 ± 1.6	39.6 ± 1.1
25	63.3 ± 2.0	32.7 ± 0.9
26	62.0 ± 0.6	37.2 ± 0.3
27	59.9 ± 9.3	30.0 ± 5.0
28	58.4 ± 3.0	36.5 ± 0.7
29	59.7 ± 2.6	32.9 ± 1.3
30	59.3 ± 4.3	31.9 ± 2.3
31	61.5 ± 4.8	29.3 ± 0.4
32	62.4 ± 1.1	32.2 ± 1.6
33	59.9 ± 0.2	28.5 ± 0.1
34	60.7 ± 6.4	33.7 ± 3.8
35	60.1 ± 3.1	30.1 ± 1.6
36	64.8 ± 18.4	38.1 ± 4.3
37	59.0 ± 2.9	28.3 ± 0.7
38	61.2 ± 9.2	38.3 ± 6.1
39	60.3 ± 5.5	33.4 ± 3.7
40	55.6 ± 7.1	32.0 ± 3.4
41	53.8 ± 11.9	32.1 ± 7.0
42	52.5 ± 7.2	30.0 ± 4.5
43	58.1 ± 6.8	28.2 ± 4.3
44	57.1 ± 3.6	32.1 ± 1.8
45	55.7 ± 2.0	34.2 ± 1.1

a Soxhlet's conditions: 6 h at 70 °C.

b UAE's conditions: 15 min at 25 °C for three times.

c Average ± standard deviation from three repetitions for each of the 45 samples.

The pistachio oil yield using two pressing systems of extraction, screw press and hydraulic press, was compared in a study of Rabadán et al. [41]. The results showed moderate differences in oil yields obtained by the different presses. By using the screw press, oil yield reached 34.8% while the hydraulic press yield was 31.0% [41]. Another evaluation of nut oil yield was conducted for almond where the variable factors involved were five commercial cultivars, three consecutive growing seasons and across five sites. Wide variability for almond oil content was observed with the genotype being the main variability source. Late ripening cultivars with higher fruit development periods were found to have higher oil content. Drier harvest seasons and sites showed the best scores of oil content [42]. The variance of almond oil content among sites was in agreement with other research works. These differences could be possibly explained by pedoclimatic conditions varying from an environment to another [43].

### 3.2 Soxhlet

**Fig. 1:**
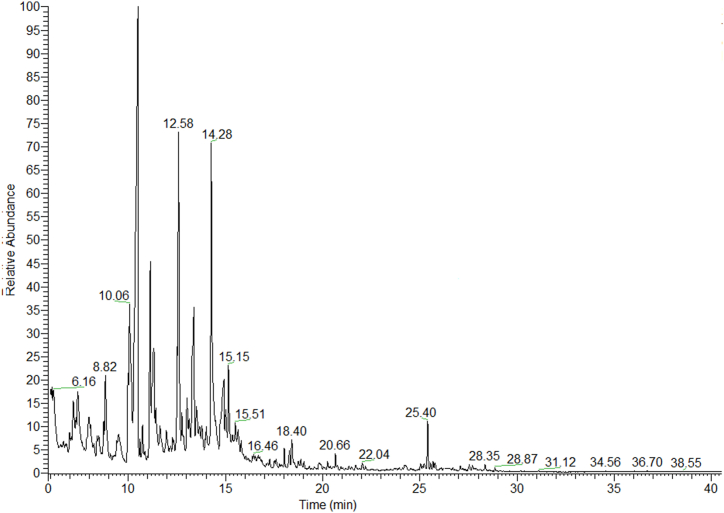
Representative chromatogram of PKO volatile components from Soxhlet.

A representative chromatogram of the PKO volatile compounds from Soxhlet is shown in Fig. 1. The composition of the identified volatiles (expressed as percentage of total volatile compounds) is presented in Table 2, Table 3, Table 4. The analysis of volatile compounds resulted in 30 components, but the main were found to be decane, nonanal and (*E*)-2-decenal. Decane gives the typical alkane odor, while the odors of fat and citrus are due to nonanal [44] which has been found in pistachio oil from various regions, such as France, Austria [45] and California [46]. As for (*E*)-2-decenal, it is a volatile compound responsible for the fatty odor of pistachio oil [44].

**Table 2:** Composition of volatile compounds, expressed as percentage (%) of total volatiles, by Soxhlet (part 1).

Samples Volatiles	1	2	3	4	5	6	7	8	9	10	11	12	13	14	15
Diacetone alcohol	5.7 ± 0.0^a^	8.0 ± 0.6	1.5 ± 0.6	6.7 ± 2.2	6.8 ± 1.7	6.8 ± 0.0	1.5 ± 0.9	3.3 ± 2.1	3.5 ± 0.6	8.0 ± 1.2	4.1 ± 0.6	1.5 ± 0.1	6.7 ± 2.5	2.5 ± 0.0	12.1 ± 4.4
ο-Xylene	–	–	–	–	–	–	–	–	–	–	5.9 ± 2.8	–	9.8 ± 0.8	–	–
Styrene	–	–	–	–	–	–	–	–	–	–	–	–	–	1.5 ± 0.5	–
Nonane	–	–	–	–	–	–	–	6.8 ± 2.4	6.3 ± 0.8	–	14.4 ± 3.7	–	–	–	–
α-Pinene	–	–	–	–	–	–	–	–	–	–	–	–	–	5.3 ± 2.4	–
Camphene	–	–	4.7 ± 1.5	–	2.8 ± 0.6	–	16.7 ± 0.5	–	–	5.3 ± 0.2	–	16.6 ± 0.1	10.8 ± 1.6	21.9 ± 1.2	–
β-Pinene	–	–	–	–	–	–	–	–	–	–	–	–	–	1.3 ± 0.9	–
Octanal	–	–	–	–	–	7.1 ± 0.1	–	–	–	–	–	–	–	–	2.3 ± 0.5
β-Myrcene	–	–	9.3 ± 1.7	4.6 ± 1.3	2.2 ± 0.7	–	14.1 ± 2.8	–	–	–	–	14.0 ± 0.1	–	32.5 ± 3.4	–
Decane	44.2 ± 1.3	–	6.6 ± 1.2	65.2 ± 3.6	12.2 ± 3.4	–	9.2 ± 3.6	42.2 ± 0.4	53.3 ± 5.3	29.8 ± 2.4	65.5 ± 1.7	9.0 ± 0.0	62.3 ± 3.9	–	6.7 ± 1.7
*p*-Cymene	–	–	6.9 ± 0.4	–	–	1.0 ± 0.6	17.8 ± 5.4	–	–	–	–	17.7 ± 0.3	–	17.3 ± 0.7	–
d-Limonene	–	–	2.5 ± 1.3	–	1.0 ± 0.8	0.4 ± 0.4	7.5 ± 0.8	–	–	–	–	7.4 ± 0.5	–	8.0 ± 0.6	–
γ-Terpinene	–	–	–	–	–	–	2.4 ± 0.2	–	–	–	–	2.3 ± 0.1	–	1.5 ± 0.3	–
α-Terpinolene	–	–	–	–	–	–	–	–	–	–	–	–	–	0.3 ± 0.0	–
Linalool	–	3.4 ± 1.2	11.0 ± 0.2	1.7 ± 0.6	1.0 ± 0.0	3.5 ± 0.7	3.8 ± 1.6	1.2 ± 0.9	–	0.6 ± 0.4	5.3 ± 2.3	3.7 ± 0.1	1.3 ± 0.5	4.3 ± 1.4	18.9 ± 2.5
Undecane	44.7 ± 3.6	–	–	2.7 ± 0.7	0.6 ± 0.3	–	–	1.2 ± 2.3	2.7 ± 0.4	1.0 ± 0.8	–	–	1.7 ± 0.7	–	–
Nonanal	–	26.9 ± 2.1	15.9 ± 2.3	6.6 ± 2.1	21.3 ± 0.8	24.6 ± 1.7	6.6 ± 2.4	10.5 ± 0.4	8.9 ± 2.4	15.7 ± 4.6	–	6.4 ± 0.2	3.7 ± 0.8	1.7 ± 0.1	16.3 ± 1.3
Camphor	–	–	2.2 ± 0.0	1.3 ± 0.2	–	–	2.5 ± 0.6	–	–	–	–	2.4 ± 0.1	–	–	–
(*E*)-2-Nonenal	–	4.0 ± 0.9	–	–	3.1 ± 0.4	3.6 ± 0.3	1.1 ± 1.7	2.3 ± 1.8	1.7 ± 1.2	2.9 ± 0.8	–	1.0 ± 0.0	–	–	–
Terpinen-4-ol	–	–	1.8 ± 0.8	–	–	–	0.5 ± 0.2	–	–	–	–	0.4 ± 0.0	–	0.4 ± 0.0	1.2 ± 0.4
Dodecane	3.0 ± 0.3	0.6 ± 0.2	0.9 ± 0.1	2.4 ± 0.0	0.5 ± 0.3	0.8 ± 0.0	0.5 ± 0.0	1.9 ± 0.7	1.2 ± 0.4	1.0 ± 0.5	1.6 ± 0.4	0.5 ± 0.1	1.5 ± 0.1	–	–
Decanal	–	2.2 ± 1.7	2.0 ± 0.2	–	1.6 ± 0.5	1.7 ± 0.4	0.6 ± 0.3	1.5 ± 0.3	0.9 ± 0.2	1.6 ± 0.8	–	0.7 ± 0.0	–	–	1.2 ± 0.8
Pulegone	–	0.3 ± 0.0	1.5 ± 0.6	0.4 ± 0.0	–	–	1.1 ± 0.7	0.4 ± 0.2	–	–	0.3 ± 0.0	1.2 ± 0.1	–	0.4 ± 0.0	1.1 ± 0.0
Linalool acetate	–	1.7 ± 0.4	3.7 ± 0.4	1.0 ± 0.8	0.3 ± 0.0	0.5 ± 0.2	1.4 ± 0.5	0.7 ± 0.1	–	–	1.1 ± 0.9	1.4 ± 0.2	0.6 ± 0.0	0.8 ± 0.3	4.5 ± 0.6
(*E*)-2-Decenal	–	25.2 ± 2.6	13.1 ± 3.1	2.7 ± 1.4	21.5 ± 3.5	26.0 ± 0.8	5.4 ± 0.0	12.3 ± 2.2	9.2 ± 0.0	16.1 ± 4.1	–	5.5 ± 0.2	–	–	23.4 ± 1.1
Bornyl acetate	–	0.3 ± 0.1	2.3 ± 0.4	0.3 ± 0.0	,-	–	0.7± 0.1	–	–	–	0.5 ± 0.1	0.8 ± 0.1	0.6 ± 0.2	0.4 ± 0.0	1.9 ± 0.9
Tridecane	1.7 ± 0.1	–	0.9 ± 0.3	2.0 ± 0.9	0.6 ± 0.3	–	–	2.5 ± 0.9	1.0 ± 0.1	0.9 ± 0.6	1.1 ± 0.3	–	0.7 ± 0.3	–	–
(*E*,*E*)-2,4-Decadienal	–	3.8 ± 0.5	–	–	5.0 ± 0.4	–	1.3 ± 0.8	1.4 ± 1.0	2.1 ± 0.7	1.3 ± 0.5	–	1.5 ± 0.3	–	–	3.4 ± 0.7
(*E*)-2-Undecenal	–	23.0 ± 2.3	12.5 ± 2.4	1.5 ± 0.5	19.2 ± 3.3	23.3 ± 4.5	5.0 ± 2.3	10.6 ± 1.4	8.6 ± 2.5	15.2 ± 3.2	–	5.1 ± 0.1	–	–	6.9 ± 0.8
Tetradecane	0.7 ± 0.2	0.8 ± 0.3	0.9 ± 0.3	0.9 ± 0.6	0.5 ± 0.2	0.7 ± 0.4	0.4 ± 0.0	1.4 ± 0.3	0.7 ± 0.4	0.6 ± 0.4	0.4 ± 0.2	0.9 ± 0.1	0.3 ± 0.1	–	–

a Average ± standard deviation from three repetitions for each sample.

**Table 3:** Composition of volatile compounds, expressed as percentage (%) of total volatiles, by Soxhlet (part 2).

Samples Volatiles	16	17	18	19	20	21	22	23	24	25	26	27	28	29	30
Diacetone alcohol	–	6.4 ± 2.4	5.7 ± 1.8	–	–	–	–	–	–	–	–	5.7 ± 0.6	–	–	23.7 ± 1.7
ο-Xylene	–	–	–	–	–	–	–	–	–	–	–	–	–	–	–
Styrene	–	–	–	–	–	–	–	–	19.7 ± 2.8	–	–	–	–	–	–
Nonane	–	–	7.9 ± 1.7	14.4 ± 4.1	20.5 ± 0.3	9.0 ± 2.8	3.7 ± 2.1	–	–	13.4 ± 0.9	–	8.2 ± 1.2	16.2 ± 4.6	11.2 ± 3.1	–
α-Pinene	–	–	–	–	–	–	–	–	–	–	–	–	–	–	30.7 ± 2.5
Camphene	–	–	–	–	–	–	–	–	–	–	14.8 ± 5.6	–	–	–	11.7 ± 3.4
β-Pinene	–	–	–	–	–	–	–	–	–	–	–	–	–	–	–
Octanal	–	–	–	–	–	–	–	–	–	–	6.5 ± 0.5	–	–	–	4.1 ± 0.6
β-Myrcene	–	–	–	–	–	–	–	–	–	–	8.2 ± 3.0	–	–	–	–
Decane	54.7±7.3^a^	70.6 ± 0.9	70.8 ± 2.6	72.3 ± 5.8	68.8 ± 6.8	39.3 ± 4.1	9.8 ± 2.4	89.5 ± 1.8	53.1 ± 1.9	78.8 ± 8.9	–	76.6 ± 4.4	83.1 ± 5.2	33.6 ± 2.4	–
*p*-Cymene	–	7.6 ± 0.6	6.5 ± 2.0	2.7 ± 1.1	7.2 ± 2.4	–	–	–	–	2.0 ± 0.3	9.0 ± 2.6	2.2 ± 0.6	–	–	5.9 ± 0.0
d-Limonene	–	–	–	–	–	–	–	–	–	–	4.2 ± 1.9	–	–	–	3.3 ± 1.2
γ-Terpinene	–	–	–	–	–	–	–	–	–	–	–	–	–	–	–
α-Terpinolene	2.8 ± 0.5	2.4 ± 1.3	–	–	–	–	–	–	–	–	–	–	–	–	–
Linalool	–	–	–	1.4 ± 0.3	0.6 ± 0.3	–	–	6.1 ± 2.6	–	–	0.7 ± 0.5	–	–	40.5 ± 3.0	–
Undecane	–	3.3 ± 0.7	3.0 ± 1.8	–	2.0 ± 1.0	1.8 ± 0.9	0.7 ± 0.3	–	1.9 ± 0.6	2.9 ± 1.0	–	3.9 ± 2.1	–	2.5 ± 0.1	–
Nonanal	13.1 ± 0.7	3.0 ± 1.4	3.2 ± 0.9	6.0 ± 1.9	–	13.7 ± 2.7	24.2 ± 2.6	–	7.0 ± 2.5	1.4 ± 0.4	15.7 ± 1.7	–	–	8.8 ± 1.7	11.9 ± 4.2
Camphor	–	–	–	–	–	–	–	–	–	–	1.2 ± 0.8	–	–	–	–
(*E*)-2-Nonenal	1.8 ± 0.1	–	–	–	–	2.5 ± 1.1	4.3 ± 1.5	–	1.2 ± 0.9	–	2.6 ± 1.7	–	–	–	–
Terpinen-4-ol	–	–	–	–	–	–	–	–	–	–	–	–	–	–	–
Dodecane	1.3 ± 0.0	2.8 ± 0.9	1.4 ± 0.4	1.6 ± 0.5	0.6 ± 0.2	1.3 ± 0.6	1.2 ± 0.9	2.0 ± 0.8	0.8 ± 0.3	0.8 ± 0.5	1.0 ± 0.4	2.1 ± 1.3	–	1.4 ± 0.7	1.4 ± 0.3
Decanal	0.8 ± 0.3	–	–	–	–	1.1 ± 0.3	1.7 ± 0.5	–	0.7 ± 0.4	–	1.3 ± 0.8	–	–	1.0 ± 0.3	–
Pulegone	–	–	–	–	–	–	–	–	–	–	0.6 ± 0.0	–	–	–	–
Linalool acetate	–	–	–	–	–	–	–	–	–	–	0.6 ± 0.0	–	–	–	–
(*E*)-2-Decenal	12.2 ± 2.6	2.4 ± 1.5	0.8 ± 0.3	–	–	14.7 ± 2.5	24.6 ± 1.6	–	7.3 ± 2.9	–	15.4 ± 1.1	–	–	–	3.4 ± 2.1
Bornyl acetate	–	–	–	–	–	–	–	–	–	–	0.4 ± 0.1	–	–	–	–
Tridecane	0.7 ± 0.2	1.1 ± 0.7	0.6 ± 0.2	1.2 ± 0.6	0.4 ± 0.1	1.0 ± 0.5	1.1 ± 0.7	1.4 ± 0.6	0.6 ± 0.0	0.5 ± 0.1	0.9 ± 0.3	0.9 ± 0.5	0.8 ± 0.4	0.7 ± 0.2	1.0 ± 0.3
(*E*,*E*)-2,4-Decadienal	3.1 ± 1.0	–	–	–	–	2.0 ± 0.9	4.9 ± 2.5	–	0.6 ± 0.2	–	2.3 ± 0.6	–	–	–	–
(*E*)-2-Undecenal	9.2 ± 0.9	–	–	–	–	12.7 ± 3.4	23.0 ± 0.8	–	6.8 ± 1.5	–	14.5 ± 0.9	–	–	–	3.1 ± 0.8
Tetradecane	0.3 ± 0.1	0.4 ± 0.0	0.2 ± 0.0	0.4 ± 0.0	–	0.7 ± 0.0	0.9 ± 0.2	1.1 ± 0.7	0.3 ± 0.1	0.2 ± 0.0	0.4 ± 0.0	0.5 ± 0.0	–	0.3 ± 0.0	–

a Average ± standard deviation from three repetitions for each sample.

**Table 4:** Composition of volatile compounds, expressed as percentage (%) of total volatiles, by Soxhlet (part 3).

Samples Volatiles	31	32	33	34	35	36	37	38	39	40	41	42	43	44	45
Diacetone alcohol	–	–	–	27.8 ± 3.3	9.6 ± 2.4	–	–	–	–	3.7 ± 1.7	–	–	–	–	23.9 ± 2.7
ο-Xylene	–	–	–	–	–	–	–	–	–	–	–	–	–	–	–
Styrene	–	–	–	–	–	–	–	–	–	–	–	–	–	–	–
Nonane	6.9 ± 0.7^2^	–	13.3 ± 3.8	–	–	16.8 ± 4.1	16.8 ± 2.0	9.8 ± 0.4	17.4 ± 5.9	14.0 ± 2.8	22.7 ± 3.9	9.6 ± 2.4	8.2 ± 1.8	19.8 ± 4.3	–
α-Pinene	–	–	–	21.1 ± 0.5	–	–	–	–	–	–	–	–	–	–	–
Camphene	–	–	–	13.1 ± 2.4	–	–	–	–	–	5.9 ± 1.3	–	–	–	–	–
β-Pinene	–	–	–	–	–	–	–	–	–	–	–	–	–	–	–
Octanal	–	–	–	4.5 ± 1.3	–	–	–	–	–	–	–	–	–	–	–
β-Myrcene	–	–	–	–	–	–	–	–	–	–	–	–	–	–	–
Decane	44.9 ± 4.8	90.1 ± 4.7	82.7 ± 4.6	–	77.1 ± 6.2	49.5 ± 2.8	49.5 ± 3.6	45.0 ± 2.4	73.5 ± 2.7	53.6 ± 7.1	73.4 ± 4.5	45.2 ± 5.7	47.8 ± 1.5	53.2 ± 2.6	67.1 ± 1.9
*p*-Cymene	–	–	–	7.9 ± 0.6	–	–	–	–	–	–	–	–	–	–	–
d-Limonene	–	–	–	4.5 ± 2.1	–	–	–	–	–	–	–	–	–	–	–
γ-Terpinene	–	–	–	–	–	–	–	–	–	–	–	–	–	–	–
α-Terpinolene	–	–	–	–	–	–	–	–	–	–	–	–	–	–	–
Linalool	–	6.2 ± 2.3	–	–	–	–	–	–	–	–	–	–	–	–	–
Undecane	2.7 ± 0.2	–	–	–	4.7 ± 0.8	3.9 ± 0.7	3.9 ± 1.7	2.1 ± 0.0	–	2.9 ± 0.2	2.6 ± 0.6	1.9 ± 0.9	1.9 ± 0.7	1.7 ± 0.8	3.8 ± 1.5
Nonanal	13.3 ± 2.3	–	–	12.9 ± 0.5	–	19.2 ± 3.4	19.2 ± 2.8	11.7 ± 0.2	6.0 ± 0.4	5.4 ± 2.3	–	10.4 ± 2.8	10.8 ± 3.1	6.6 ± 3.3	–
Camphor	–	–	–	–	–	–	–	–	–	–	–	–	–	–	–
(*E*)-2-Nonenal	2.9 ± 0.6	–	–	–	–	–	–	2.4 ± 0.8	–	–	–	2.5 ± 1.1	2.7 ± 0.6	1.3 ± 0.1	–
Terpinen-4-ol	–	–	–	–	–	–	–	–	–	–	–	–	–	–	–
Dodecane	1.4 ± 0.5	2.0 ± 1.2	1.1 ± 0.3	1.7 ± 0.4	2.5 ± 0.3	2.2 ± 0.3	2.2 ± 0.0	1.1 ± 0.2	1.4 ± 0.5	1.0 ± 0.1	1.0 ± 0.9	1.2 ± 0.5	1.1 ± 0.4	0.7 ± 0.0	2.5 ± 1.0
Decanal	1.6 ± 0.9	–	–	2.3 ± 0.0	–	2.3 ± 1.2	2.3 ± 0.0	1.3 ± 0.7	–	0.9 ± 0.7	–	1.4 ± 0.7	1.5 ± 0.9	0.8 ± 0.2	–
Pulegone	–	–	–	–	–	–	–	–	–	–	–	–	–	–	–
Linalool acetate	–	–	–	–	–	–	–	–	–	–	–	–	–	–	–
(*E*)-2-Decenal	12.4 ± 1.3	–	1.7 ± 0.1	4.2 ± 0.1	–	4.4 ± 0.9	4.4 ± 2.0	12.0 ± 1.3	–	5.6 ± 0.3	–	12.3 ± 2.9	11.9 ± 1.7	7.1 ± 1.6	–
Bornyl acetate	–	–	–	–	–	–	–	–	–	–	–	–	–	–	–
Tridecane	1.1 ± 0.0	1.2 ± 0.3	0.9 ± 0.3	–	3.2 ± 0.4	1.2 ± 0.0	1.2 ± 0.6	1.3 ± 0.4	1.3 ± 0.2	0.6 ± 0.1	0.3 ± 0.1	1.1 ± 0.6	1.3 ± 0.2	0.7 ± 0.3	1.9 ± 0.8
(*E*,*E*)-2,4-Decadienal	1.4 ± 0.0	–	–	–	–	–	–	2.3 ± 1.1	–	0.5 ± 0.2	–	2.3 ± 1.2	1.8 ± 0.0	1.3 ± 0.8	–
(*E*)-2-Undecenal	10.6 ± 0.4	–	–	–	–	–	–	10.3 ± 0.1	–	5.5 ± 2.4	–	11.3 ± 0.4	10.6 ± 2.8	6.7 ± 2.4	–
Tetradecane	0.9 ± 0.2	0.5 ± 0.0	0.4 ± 0.0	–	2.9 ± 0.7	0.4 ± 0.1	0.4 ± 0.1	0.8 ± 0.0	0.5 ± 0.1	0.4 ± 0.0	–	0.8 ± 0.0	0.7 ± 0.1	0.3 ± 0.0	0.8 ± 0.0

1Average ± standard deviation from three repetitions for each sample.

### 3.3 UAE

**Fig. 2:**
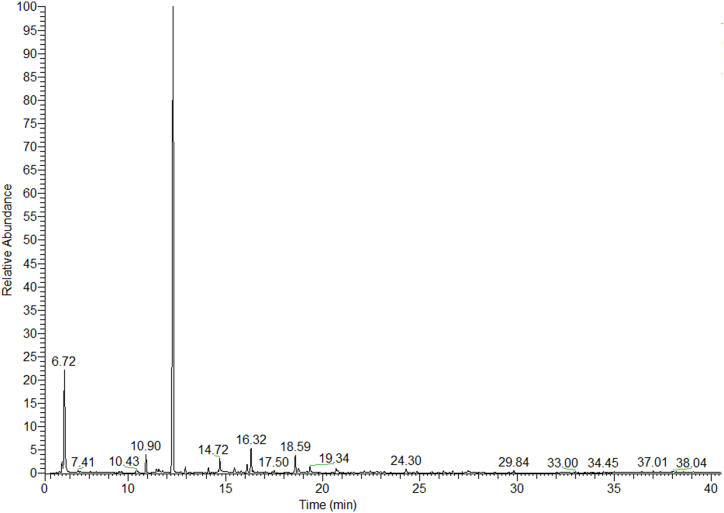
Representative chromatogram of PKO volatile components from UAE.

A representative chromatogram of the PKO volatile compounds from UAE is shown in Fig. 2. The composition of the identified volatiles (expressed as percentage of total volatile compounds) is presented in Table 5, Table 6, Table 7. Among the 34 volatile compounds identified in PKO, the major ones were α-pinene, octane and decane. In detail, α-pinene is the most important volatile compound in pistachio samples [33] and its odor bears a resemblance to pine and turpentine, while octane and decane releases an alkane odor [44]. Similar volatile components of pistachio oils extracted by HS-SPME and analyzed by GC-MS were found in other studies. d-limonene, α-pinene, 3-carene, β-myrcene, nonanal and α-terpinolene were those with the highest content [[30], [46], [47], [48]].

**Table 5:** Composition of volatile compounds, expressed as percentage (%) of total volatiles, by UAE (part 1).

Samples Volatiles	1	2	3	4	5	6	7	8	9	10	11	12	13	14	15
3-Hexanol	–	0.8 ± 0.2	0.1 ± 0.0	–	1.0 ± 0.0	0.2 ± 0.1	0.3 ± 0.0	–	–	2.9 ± 0.0	0.8 ± 0.0	–	–	0.8 ± 0.3	–
Octane	4.6 ± 0.0^a^	43.5 ± 1.6	2.4 ± 0.1	4.0 ± 0.2	13.0 ± 0.7	5.3 ± 3.4	5.4 ± 0.0	6.2 ± 0.0	4.8 ± 0.1	50.6 ± 0.0	31.4 ± 1.7	39.5 ± 0.0	36.1 ± 1.8	20.4 ± 0.7	1.2 ± 0.0
ο-Xylene	–	–	0.1 ± 0.0	3.7 ± 0.3	–	–	–	0.8 ± 0.0	–	–	–	–	–	–	–
Styrene	21.8 ± 2.7	–	0.2 ± 0.0	12.5 ± 0.7	0.6 ± 0.1	–	–	0.5 ± 0.0	–	–	–	–	5.4 ± 0.8	–	–
Nonane	–	8.8 ± 0.1	0.2 ± 0.0	0.9 ± 0.0	2.0 ± 0.1	1.4 ± 0.1	1.8 ± 0.0	0.9 ± 0.0	1.3 ± 0.0	4.0 ± 0.0	5.7 ± 0.1	9.1 ± 0.0	3.5 ± 0.2	3.8 ± 0.2	0.7 ± 0.0
Tricyclene	–	–	0.4 ± 0.0	–	0.2 ± 0.0	0.4 ± 0.1	0.3 ± 0.0	0.3 ± 0.0	0.6 ± 0.0	–	–	–	–	–	0.2 ± 0.0
α-Pinene	34.7 ± 1.6	14.9 ± 0.9	85.0 ± 0.6	25.2 ± 0.2	71.4 ± 0.8	82.5 ± 3.0	77.2 ± 0.0	80.1 ± 0.0	80.6 ± 0.6	26.9 ± 0.0	42.4 ± 1.2	18.0 ± 0.0	40.4 ± 0.3	54.6 ± 0.2	69.0 ± 0.0
Camphene	7.3 ± 0.5	–	1.2 ± 0.0	8.3 ± 0.3	0.7 ± 0.0	1.1 ± 0.0	1.2 ± 0.0	0.8 ± 0.0	1.3 ± 0.0	0.4 ± 0.0	–	–	0.6 ± 0.1	0.8 ± 0.1	0.7 ± 0.0
β-Pinene	1.2 ± 0.3	–	1.1 ± 0.0	1.4 ± 0.1	0.6 ± 0.0	0.7 ± 0.0	0.7 ± 0.0	0.7 ± 0.0	0.9 ± 0.1	0.4 ± 0.0	–	–	0.7 ± 0.1	–	0.8 ± 0.0
β-Myrcene	–	–	1.9 ± 0.1	7.6 ± 0.1	1.4 ± 0.0	0.8 ± 0.1	1.0 ± 0.0	1.7 ± 0.0	1.3 ± 0.1	2.6 ± 0.0	–	–	–	–	4.5 ± 0.0
Decane	1.1 ± 0.3	26.2 ± 1.3	–	1.7 ± 0.6	–	3.8 ± 0.2	5.6 ± 0.0	–	4.3 ± 0.2	–	16.1 ± 0.5	27.7 ± 0.0	–	10.7 ± 0.4	–
2-Carene	1.3 ± 0.1	–	0.9 ± 0.1	1.4 ± 0.1	0.5 ± 0.0	0.4 ± 0.1	0.5 ± 0.0	0.5 ± 0.0	0.5 ± 0.1	0.5 ± 0.0	–	–	0.6 ± 0.0	–	1.0 ± 0.0
3-Carene	0.4 ± 0.1	–	0.2 ± 0.1	0.6 ± 0.1	0.1 ± 0.0	–	–	0.1 ± 0.0	–	0.2 ± 0.0	–	–	–	–	–
α-Terpinene	–	–	0.2 ± 0.0	–	–	–	–	–	–	–	–	–	0.2 ± 0.2	–	–
*p*-Cymene	3.4 ± 0.2	–	0.6 ± 0.1	4.4 ± 0.0	1.0 ± 0.0	1.1 ± 0.0	1.6 ± 0.0	0.7 ± 0.0	1.3 ± 0.1	1.2 ± 0.0	–	–	1.9 ± 0.4	–	2.6 ± 0.0
d-Limonene	5.9 ± 0.4	2.6 ± 0.2	4.5 ± 0.3	5.5 ± 0.1	2.7 ± 0.0	1.7 ± 0.2	2.5 ± 0.0	2.3 ± 0.0	2.1 ± 0.2	3.6 ± 0.0	2.1 ± 0.2	–	3.1 ± 0.3	6.3 ± 0.2	6.1 ± 0.0
*cis*-β-Ocimene	–	–	0.1 ± 0.0	0.5 ± 0.1	–	–	–	–	–	–	–	–	–	–	0.2 ± 0.0
*trans*-β-Ocimene	–	–	–	0.8 ± 0.3	0.1 ± 0.0	–	–	–	–	0.2 ± 0.0	–	–	–	–	0.2 ± 0.0
γ-Terpinene	1.0 ± 0.2	–	0.2 ± 0.0	0.9 ± 0.3	0.2 ± 0.0	–	–	0.2 ± 0.0	–	0.4 ± 0.0	–	–	0.1 ± 0.2	1.1 ± 0.1	0.7 ± 0.0
α-Terpinolene	8.5 ± 0.8	0.1 ± 0.0	0.4 ± 0.0	9.3 ± 0.0	2.0 ± 0.0	0.1 ± 0.0	0.2 ± 0.0	0.9 ± 0.0	0.1 ± 0.0	2.3 ± 0.0	0.3 ± 0.1	–	2.6 ± 0.1	0.2 ± 0.0	1.8 ± 0.0
*p*-Cymenene	2.3 ± 0.1	–	–	3.7 ± 0.6	0.5 ± 0.0	0.1 ± 0.0	0.2 ± 0.0	0.3 ± 0.0	0.1 ± 0.0	0.5 ± 0.0	–	–	–	–	1.3 ± 0.0
Linalool	–	–	–	–	–	–	0.6 ± 0.0	0.1 ± 0.0	–	0.1 ± 0.0	–	–	–	–	0.5 ± 0.0
Undecane	–	0.3 ± 0.3	–	–	–	0.1 ± 0.0	–	–	0.1 ± 0.1	–	–	1.2 ± 0.0	–	–	–
Nonanal	–	–	–	–	0.7 ± 0.0	–	0.2 ± 0.0	0.9 ± 0.0	0.2 ± 0.0	0.8 ± 0.0	–	1.3 ± 0.0	–	–	1.3 ± 0.0
*p*-Cymen-8-ol	1.5 ± 0.1	0.1 ± 0.0	–	1.6 ± 0.4	0.3 ± 0.0	–	–	0.3 ± 0.0	0.1 ± 0.0	0.4 ± 0.0	–	–	–	–	0.8 ± 0.0
Octanoic acid, ethyl ester	–	0.5 ± 0.1	–	–	–	0.1 ± 0.0	0.3 ± 0.0	0.4 ± 0.0	–	–	0.3 ± 0.0	0.7 ± 0.0	–	0.4 ± 0.1	2.3 ± 0.0
Dodecane	1.3 ± 0.2	0.4 ± 0.0	–	0.8 ± 0.1	0.1 ± 0.0	0.1 ± 0.0	0.1 ± 0.0	0.3 ± 0.0	0.1 ± 0.0	–	0.3 ± 0.0	0.9 ± 0.0	1.0 ± 0.0	0.2 ± 0.0	0.3 ± 0.0
Pulegone	0.9 ± 0.1	0.4 ± 0.0	–	0.4 ± 0.5	0.5 ± 0.0	0.1 ± 0.0	0.2 ± 0.0	0.3 ± 0.0	0.1 ± 0.0	0.7 ± 0.0	0.2 ± 0.1	0.5 ± 0.0	1.4 ± 0.0	0.2 ± 0.1	2.0 ± 0.0
Bornyl acetate	0.4 ± 0.0	–	–	1.3 ± 0.1	–	–	–	0.2 ± 0.0	–	0.8 ± 0.0	–	–	–	–	0.4 ± 0.0
2-Undecanone	0.8 ± 0.0	0.2 ± 0.2	–	1.3 ± 0.2	0.2 ± 0.0	–	–	0.2 ± 0.0	–	0.3 ± 0.0	0.1 ± 0.1	–	–	–	–
Tridecane	–	0.4 ± 0.0	–	–	–	0.1 ± 0.0	0.1 ± 0.0	0.3 ± 0.0	0.1 ± 0.0	–	0.2 ± 0.0	0.6 ± 0.0	0.7 ± 0.0	0.2 ± 0.0	0.3 ± 0.0
Decanoic acid, ethyl ester	–	0.2 ± 0.0	–	–	–	–	–	0.1 ± 0.0	–	–	–	–	–	0.1 ± 0.0	0.5 ± 0.0
Tetradecane	0.7 ± 0.0	0.4 ± 0.0	–	0.6 ± 0.1	0.2 ± 0.0	0.1 ± 0.0	0.1 ± 0.0	0.2 ± 0.0	0.1 ± 0.0	0.3 ± 0.0	0.2 ± 0.0	0.5 ± 0.0	0.8 ± 0.3	0.2 ± 0.0	0.6 ± 0.0
Phthalic acid, butyl octyl ester	1.2 ± 0.5	0.2 ± 0.1	–	1.6 ± 0.2	0.1 ± 0.0	–	–	0.1 ± 0.0	0.1 ± 0.0	0.1 ± 0.0	0.1 ± 0.0	0.2 ± 0.0	0.9 ± 0.1	0.1 ± 0.0	0.1 ± 0.0

a Average ± standard deviation from three repetitions for each sample.

**Table 6:** Composition of volatile compounds, expressed as percentage (%) of total volatiles, by UAE (part 2).

Samples Volatiles	16	17	18	19	20	21	22	23	24	25	26	27	28	29	30
3-Hexanol	–	1.6 ± 0.5	–	–	1.9 ± 0.0	–	1.6 ± 0.2	–	–	1.6 ± 0.2	2.0 ± 0.5	2.6 ± 0.0	2.6 ± 0.0	–	2.6 ± 0.0
Octane	53.0 ± 0.0^a^	36.8 ± 0.4	49.3 ± 0.6	48.2 ± 0.7	43.8 ± 0.0	2.2 ± 0.2	56.9 ± 0.4	15.0 ± 0.2	36.2 ± 0.0	46.6 ± 3.3	47.5 ± 3.1	43.4 ± 0.0	46.8 ± 0.0	50.1 ± 0.6	46.8 ± 0.0
ο-Xylene	–	0.6 ± 0.1	–	–	–	8.8 ± 0.3	–	–	4.8 ± 0.0	–	–	–	–	–	–
Styrene	–	–	–	–	–	–	–	–	–	–	–	–	–	–	–
Nonane	4.9 ± 0.0	3.4 ± 0.1	10.5 ± 0.3	12.4 ± 2.8	9.4 ± 0.0	–	–	3.4 ± 0.0	3.8 ± 0.0	9.0 ± 0.3	8.5 ± 0.1	9.4 ± 0.0	9.6 ± 0.0	6.6 ± 0.0	9.6 ± 0.0
Tricyclene	–	–	–	–	–	–	–	–	–	–	–	–	–	–	–
α-Pinene	14.9 ± 0.0	43.3 ± 1.0	–	8.5 ± 0.2	7.6 ± 0.0	41.0 ± 0.8	21.2 ± 0.6	63.2 ± 0.3	18.5 ± 0.0	10.5 ± 0.6	12.4 ± 4.5	6.3 ± 0.0	9.9 ± 0.0	30.9 ± 0.1	9.9 ± 0.0
Camphene	0.6 ± 0.0	0.6 ± 0.1	–	–	–	1.3 ± 0.1	–	1.4 ± 0.0	1.0 ± 0.0	–	–	–	–	0.4 ± 0.0	–
β-Pinene	0.9 ± 0.0	0.7 ± 0.1	–	–	–	1.4 ± 0.1	–	–	1.6 ± 0.0	–	–	–	–	0.9 ± 0.1	–
β-Myrcene	–	–	–	–	–	6.0 ± 0.1	–	–	–	–	–	–	–	–	–
Decane	–	–	31.8 ± 0.5	24.6 ± 1.8	28.8 ± 0.0	0.6 ± 0.1	–	11.4 ± 0.4	0.8 ± 0.0	27.2 ± 2.1	24.4 ± 1.2	33.3 ± 0.0	29.1 ± 0.0	–	29.1 ± 0.0
2-Carene	–	–	–	–	–	2.1 ± 0.0	–	–	1.4 ± 0.0	–	–	–	–	0.5 ± 0.0	–
3-Carene	1.1 ± 0.0	1.0 ± 0.1	–	–	–	–	0.6 ± 0.4	–	0.4 ± 0.0	–	–	–	–	–	–
α-Terpinene	–	–	–	–	–	0.6 ± 0.0	–	–	–	–	–	–	–	–	–
*p*-Cymene	0.8 ± 0.0	0.7 ± 0.0	–	–	–	2.6 ± 0.1	0.6 ± 0.0	–	2.7 ± 0.0	–	–	–	–	0.6 ± 0.0	–
d-Limonene	1.5 ± 0.0	2.2 ± 0.1	–	–	–	5.8 ± 0.0	1.0 ± 0.0	2.7 ± 0.2	2.7 ± 0.0	–	–	–	–	1.3 ± 0.1	–
*cis*-β-Ocimene	–	–	–	–	–	0.3 ± 0.1	–	–	–	–	–	–	–	–	–
*trans*-β-Ocimene	–	–	–	–	–	0.6 ± 0.1	–	–	–	–	–	–	–	–	–
γ-Terpinene	–	–	–	–	–	0.6 ± 0.0	–	–	–	–	–	–	–	–	–
α-Terpinolene	11.8 ± 0.0	2.0 ± 0.3	1.2 ± 0.1	2.1 ± 0.0	1.4 ± 0.0	14.1 ± 0.2	8.8 ± 0.2	0.3 ± 0.0	5.8 ± 0.0	1.3 ± 0.5	2.2 ± 0.3	–	–	3.1 ± 0.3	–
*p*-Cymenene	3.0 ± 0.0	2.1 ± 0.5	–	1.7 ± 0.3	1.7 ± 0.0	3.3 ± 0.5	1.7 ± 0.4	–	10.1 ± 0.0	0.7 ± 0.2	0.8 ± 0.0	2.3 ± 0.0	–	1.0 ± 0.2	–
Linalool	–	–	–	–	–	–	–	–	–	–	–	–	–	–	–
Undecane	–	–	1.2 ± 0.0	–	1.5 ± 0.0	–	–	0.5 ± 0.0	–	–	–	–	–	–	–
Nonanal	1.1 ± 0.0	–	1.4 ± 0.1	–	–	–	–	0.6 ± 0.1	–	–	–	–	–	–	–
*p*-Cymen-8-ol	–	2.5 ± 0.2	0.5 ± 0.0	–	–	1.3 ± 0.2	–	0.1 ± 0.0	5.4 ± 0.0	–	0.7 ± 0.1	–	–	–	–
Octanoic acid, ethyl ester	–	–	–	–	–	–	–	–	–	–	–	–	–	–	–
Dodecane	2.5 ± 0.0	0.6 ± 0.0	1.5 ± 0.2	1.2 ± 0.0	2.2 ± 0.0	2.3 ± 0.0	2.8 ± 0.2	0.6 ± 0.0	1.4 ± 0.0	1.1 ± 0.2	0.4 ± 0.0	1.1 ± 0.0	0.7 ± 0.0	1.2 ± 0.1	0.7 ± 0.0
Pulegone	–	–	–	–	–	0.5 ± 0.0	–	–	–	–	–	–	–	–	–
Bornyl acetate	0.5 ± 0.0	0.3 ± 0.0	–	–	–	0.4 ± 0.0	0.4 ± 0.0	–	0.6 ± 0.0	–	–	–	–	–	–
2-Undecanone	–	0.2 ± 0.3	0.6 ± 0.1	–	–	1.2 ± 0.1	1.2 ± 0.2	–	–	–	–	–	–	–	–
Tridecane	2.2 ± 0.0	0.9 ± 0.1	1.1 ± 0.2	0.8 ± 0.0	1.2 ± 0.0	2.1 ± 0.1	2.4 ± 0.0	0.5 ± 0.0	1.7 ± 0.0	1.3 ± 0.1	0.5 ± 0.1	1.0 ± 0.0	0.8 ± 0.0	1.8 ± 0.2	0.8 ± 0.0
Decanoic acid, ethyl ester	–	–	–	–	–	–	–	–	–	–	–	–	–	–	–
Tetradecane	0.7 ± 0.0	0.4 ± 0.1	0.6 ± 0.1	0.4 ± 0.0	0.5 ± 0.0	0.7 ± 0.1	0.5 ± 0.0	0.3 ± 0.0	1.1 ± 0.0	0.5 ± 0.0	0.3 ± 0.0	0.5 ± 0.0	0.6 ± 0.0	0.8 ± 0.0	0.6 ± 0.0
Phthalic acid, butyl octyl ester	0.3 ± 0.0	0.3 ± 0.1	0.4 ± 0.1	0.1 ± 0.0	–	0.5 ± 0.2	0.4 ± 0.1	0.1 ± 0.0	–	0.2 ± 0.0	0.3 ± 0.1	–	–	0.8 ± 0.2	–

a Average ± standard deviation from three repetitions for each sample.

**Table 7:** Composition of volatile compounds, expressed as percentage (%) of total volatiles, by UAE (part 3).

Samples Volatiles	31	32	33	34	35	36	37	38	39	40	41	42	43	44	45
3-Hexanol	–	1.4 ± 0.1	0.3 ± 0.0	1.5 ± 0.0	0.6 ± 0.0	0.5 ± 0.0	1.4 ± 0.5	0.6 ± 0.0	1.6 ± 0.3	1.3 ± 0.2	–	–	1.6 ± 0.0	–	–
Octane	47.2±0.0^a^	45.8 ± 1.0	3.9 ± 0.0	52.9 ± 3.0	17.4 ± 0.0	13.5 ± 0.0	47.1 ± 4.8	8.3 ± 0.0	40.0 ± 1.1	44.5 ± 2.6	7.4 ± 0.9	46.1 ± 0.1	44.2 ± 1.5	4.0 ± 0.0	27.2 ± 0.0
ο-Xylene	2.8 ± 0.0	4.4 ± 0.2	–	–	–	–	–	–	–	–	7.3 ± 2.9	2.2 ± 0.1	–	7.3 ± 0.0	3.5 ± 0.0
Styrene	–	–	–	–	–	–	–	–	–	–	–	–	–	–	–
Nonane	6.3 ± 0.0	4.8 ± 0.0	1.1 ± 0.0	11.3 ± 1.6	3.3 ± 0.0	2.5 ± 0.0	9.0 ± 0.5	1.1 ± 0.0	8.8 ± 0.0	9.3 ± 0.1	–	5.3 ± 0.3	9.0 ± 0.4	–	3.7 ± 0.0
Tricyclene	–	–	–	–	–	–	–	–	–	–	–	–	–	–	–
α-Pinene	32.0 ± 0.0	12.0 ± 0.0	1.5 ± 0.0	–	65.2 ± 0.0	67.9 ± 0.0	14.6 ± 7.9	3.1 ± 0.0	19.2 ± 0.4	17.4 ± 2.3	39.5 ± 0.6	31.6 ± 0.7	15.5 ± 2.4	39.9 ± 0.0	55.9 ± 0.0
Camphene	–	–	0.3 ± 0.0	–	–	1.6 ± 0.0	–	0.2 ± 0.0	–	–	2.6 ± 1.7	0.7 ± 0.0	–	0.5 ± 0.0	0.7 ± 0.0
β-Pinene	–	0.5 ± 0.1	0.9 ± 0.0	–	–	–	–	0.8 ± 0.0	–	–	1.6 ± 0.1	1.1 ± 0.0	–	–	0.8 ± 0.0
β-Myrcene	–	–	2.4 ± 0.0	–	–	2.2 ± 0.0	–	2.2 ± 0.0	–	–	–	–	–	–	–
Decane	–	0.6 ± 0.0	3.0 ± 0.0	30.6 ± 1.4	8.8 ± 0.0	7.8 ± 0.0	26.7 ± 2.0	0.1 ± 0.0	27.1 ± 0.6	25.4 ± 0.4	1.1 ± 0.2	0.2 ± 0.1	27.0 ± 0.5	0.5 ± 0.0	0.2 ± 0.0
2-Carene	–	0.9 ± 0.0	–	–	–	–	–	0.2 ± 0.0	–	–	1.4 ± 0.2	–	–	1.2 ± 0.0	0.6 ± 0.0
3-Carene	–	–	–	–	–	–	–	–	–	–	0.4 ± 0.0	0.5 ± 0.0	–	0.4 ± 0.0	–
α-Terpinene	–	–	–	–	–	–	–	0.2 ± 0.0	–	–	–	–	–	–	–
*p*-Cymene	1.1 ± 0.0	1.1 ± 0.0	6.5 ± 0.0	–	–	–	–	3.1 ± 0.0	–	–	4.3 ± 1.1	0.6 ± 0.1	–	3.9 ± 0.0	0.7 ± 0.0
d-Limonene	1.1 ± 0.0	1.1 ± 0.0	72.9 ± 0.0	–	2.1 ± 0.0	2.5 ± 0.0	–	66.9 ± 0.0	–	–	7.0 ± 0.5	6.6 ± 0.3	–	8.7 ± 0.0	2.4 ± 0.0
*cis*-β-Ocimene	–	–	–	–	–	–	–	–	–	–	–	–	–	–	–
*trans*-β-Ocimene	–	–	–	–	–	–	–	0.2 ± 0.0	–	–	–	–	–	–	–
γ-Terpinene	–	–	4.4 ± 0.0	–	–	–	–	5.4 ± 0.0	–	–	–	–	–	–	–
α-Terpinolene	3.6 ± 0.0	8.2 ± 0.5	0.5 ± 0.0	0.3 ± 0.3	0.6 ± 0.0	0.5 ± 0.0	–	1.2 ± 0.0	–	0.6 ± 0.1	7.9 ± 1.3	3.4 ± 0.1	–	5.6 ± 0.0	0.9 ± 0.0
*p*-Cymenene	–	4.7 ± 0.0	2.0 ± 0.0	–	0.4 ± 0.0	0.3 ± 0.0	–	2.8 ± 0.0	–	–	4.6 ± 0.1	–	–	2.5 ± 0.0	1.4 ± 0.0
Linalool	–	–	–	–	–	–	–	–	–	–	–	–	–	–	–
Undecane	–	–	–	0.5 ± 0.5	0.5 ± 0.0	0.3 ± 0.0	0.5 ± 0.1	–	0.6 ± 0.0	–	–	–	0.6 ± 0.1	–	–
Nonanal	–	–	–	–	–	–	–	0.8 ± 0.0	1.0 ± 0.1	–	–	–	0.8 ± 0.0	4.6 ± 0.0	–
*p*-Cymen-8-ol	–	2.8 ± 0.1	–	–	0.1 ± 0.0	–	–	0.5 ± 0.0	–	–	2.7 ± 0.6	–	–	1.6 ± 0.0	–
Octanoic acid, ethyl ester	–	–	0.2 ± 0.0	–	–	–	–	1.7 ± 0.0	–	–	–	–	–	1.7 ± 0.0	–
Dodecane	1.7 ± 0.0	6.6 ± 0.4	–	0.9 ± 0.1	0.2 ± 0.0	0.2 ± 0.0	–	0.1 ± 0.0	0.6 ± 0.1	0.4 ± 0.0	1.8 ± 0.0	0.8 ± 0.0	0.5 ± 0.0	2.5 ± 0.0	0.8 ± 0.0
Pulegone	–	–	–	–	–	–	–	–	–	–	–	–	–	3.0 ± 0.0	–
Bornyl acetate	–	0.3 ± 0.0	–	–	–	–	–	–	–	–	1.1 ± 0.3	0.4 ± 0.0	–	0.7 ± 0.0	0.2 ± 0.0
2-Undecanone	1.3 ± 0.0	–	–	–	0.2 ± 0.0	–	–	0.3 ± 0.0	–	–	5.0 ± 2.9	–	–	3.8 ± 0.0	–
Tridecane	1.9 ± 0.0	3.8 ± 0.2	–	1.0 ± 0.2	0.3 ± 0.0	0.2 ± 0.0	0.4 ± 0.1	0.2 ± 0.0	0.7 ± 0.1	0.6 ± 0.1	1.2 ± 0.3	0.6 ± 0.2	0.5 ± 0.1	4.4 ± 0.0	1.1 ± 0.0
Decanoic acid, ethyl ester	–	–	–	–	–	–	–	–	–	–	–	–	–	–	–
Tetradecane	1.1 ± 0.0	0.9 ± 0.2	–	0.7 ± 0.3	0.2 ± 0.0	0.1 ± 0.0	0.4 ± 0.0	0.2 ± 0.0	0.5 ± 0.1	0.3 ± 0.1	2.3 ± 0.5	–	0.4 ± 0.0	2.1 ± 0.0	–
Phthalic acid, butyl octyl ester	–	0.3 ± 0.0	–	0.3 ± 0.1	0.1 ± 0.0	–	–	–	0.1 ± 0.0	0.2 ± 0.1	0.8 ± 0.6	–	0.1 ± 0.1	1.1 ± 0.0	–

a Average ± standard deviation from three repetitions for each sample.

The comparison of the average for each volatile component between the two types of extraction is presented in Table 8. Volatiles were categorized based on their chemical class in alcohols, hydrocarbons, benzene derivatives, cyclic monoterpenes, monoterpenes, acyclic monoterpenes, oxygenated monoterpenes, aldehydes, ketones and esters. It is evident that the most abundant volatile compounds of PKO by Soxhlet extraction constituted up to 62% of the total concentration of volatiles and the rest were minor constituents. The highest contributors to the volatile profile of PKO by UAE accounted for 75% of total volatiles’ concentration.

**Table 8:** Average (%) for each volatile component for the two types of extraction.

Volatile compounds	KRI (bibliography)	References	Average (%)	
			Types of extraction	
			Soxhlet	UAE
**Alcohols**				
3-Hexanol	797	[53]	-a	0.8
**Hydrocarbons**				
Octane	800	[53]	–	29.8
Nonane	900	[53]	6.4	4.9
Decane	1000	[22,30,39,46,[53], [54], [55], [56]]	45.8	11.1
Undecane	1100	[55]	2.4	0.2
Dodecane	1200	[53]	1.3	1.0
Tridecane	1300	[53,55]	0.9	0.9
Tetradecane	1400	[53,55]	0.5	0.5
**Benzene derivatives**				
ο-Xylene	887	[22]	0.3	1.0
Styrene	893	[45,53]	0.5	0.9
**Cyclic monoterpenes**				
Tricyclene	925	[53]	–	0.1
2-Carene	1001	[22,30,45,46,[53], [54], [55]]	–	0.4
3-Carene	1011	[22,30,45,46,[53], [54], [55]]	–	0.1
*p*-Cymene	1022	[22,30,45,53,55]	2.5	1.1
d-Limonene	1030	[22,30,39,45,46,[53], [54], [55], [56]]	0.9	5.3
α-Terpinolene	1088	[22,30,39,46,[53], [54], [55]]	0.1	2.6
*p*-Cymenene	1090	[53]	–	1.2
**Monoterpenes**				
α-Pinene	937	[22,30,39,46,[53], [54], [55], [56]]	1.3	33.7
Camphene	952	[22,30,[53], [54], [55]]	2.8	0.8
β-Pinene	979	[22,30,39,45,46,[53], [54], [55], [56]]	0.0	0.5
α-Terpinene	1017	[30,[53], [54], [55]]	–	0.0
γ-Terpinene	1060	[30,[53], [54], [55]]	0.1	0.3
Pulegone	1237	[53]	0.2	0.3
**Acyclic monoterpenes**				
β-Myrcene	991	[30,46,[53], [54], [55], [56]]	1.9	0.8
*cis*-β-Ocimene	1038	[22,45,[53], [54], [55]]	–	0.0
*trans*-β-Ocimene	1049	[22,45,[53], [54], [55]]	–	0.0
**Oxygenated monoterpenes**				
Linalool	1099	[54,55]	2.6	0.0
Camphor	1142	[53]	0.2	–
Terpinen-4-ol	1177	[53]	0.1	–
*p*-Cymen-8-ol	1183	[22,54,56]	–	0.5
Linalool acetate	1257	[53]	0.4	–
Bornyl acetate	1285	[[53], [54], [55], [56]]	0.2	0.2
**Aldehydes**				
Octanal	998	[53]	0.5	–
Nonanal	1104	[30,46,[53], [54], [55], [56]]	8.6	0.3
(*E*)-2-Nonenal	1162	[53]	1.0	–
Decanal	1206	[53]	0.8	–
(*E*)-2-Decenal	1263	[53]	7.0	–
(*E*,*E*)-2,4-Decadienal	1317	[53]	0.9	–
(*E*)-2-Undecenal	1367	[53]	5.7	–
**Ketones**				
Diacetone alcohol	838	[53]	4.1	–
2-Undecanone	1294	[53]	–	0.4
**Esters**				
Octanoic acid, ethyl ester	1196	[53]	–	0.2
Decanoic acid, ethyl ester	1396	[55]	–	0.0
Phthalic acid, butyl octyl ester	2317	[55]	–	0.2

a Not detected.

Alcohols are generally not considered to be important contributors to the flavors of oils due to their relatively high odor threshold values [48]. Most hydrocarbons and especially decane increased when utilizing Soxhlet except for octane which was missing from the Soxhlet's group of volatiles. This is in accordance with a study by Yang, Chao, Wu, Ye and Chen [49] which showed that dodecane and undecane were primary components of peanut oil at high temperatures. In addition, the levels of benzene derivatives decreased with Soxhlet extraction. Ling et al. [46] also found that styrene reduced due to high temperature (20 min at 160 °C). Indicator of the high temperature's impact on volatiles was the diminishing effect on terpenes apart from *p*-cymene, camphene, β-myrcene, linalool and linalool acetate. Similar results were also observed [33,46,50]. Gogus et al. [50] observed that the composition (type and concentration) of the major volatile constituents changed as the exposure time of the *Pistacia terebinthus* L. nut in high temperature increased. Especially, α-pinene showed a remarkable ascending percentage change from Soxhlet to UAE, while the concentration of furans, furanones, pyridines and benzene derivatives sharply increased. Research by Ling et al. [46] highlighted differences in *Pistacia vera* L. ‘Kerman’ cultivar volatile compounds between raw and thermal-treated kernels. The levels of terpenes' concentrations decreased with high temperature, but pyrazines, furans and pyrroles were present only in thermal-treated samples. Valdés García et al. [33] confirmed that heat treatment leads to formation of new compounds, disappearance or reduction of some and increase of others. As for aldehydes' class, they are generally described as green, tallow, metallic or rancid flavors [51]. In the present research, all of them appeared for the first time after analysis of PKO derived from Soxhlet, excepting nonanal which was already present with the use of UAE but reduced in comparison to Soxhlet's extraction. It was found and confirmed by other studies of the scientific community that the characteristic volatile compounds that were directly linked to an oxidized flavor were octanal, nonanal, (E)-2-nonenal, hexanal, (E)-2-heptenal, 2-octenal, (E)-2-decenal, (2E,4Z)-decadienal, (2E,4E)-decadienal and benzaldehyde [33,49,52]. These results may be explained by the heating intensity developed in UAE conditions which was lower than those of Soxhlet's. It is agreed that long time processing under high temperature is a key parameter for the formation of roasted compounds [30].

## 4 Conclusions

Food odor and aroma have a great influence on consumers' preference. These attributes are related to different volatile compounds. In this research, volatile compounds of pistachio oils from the Greek cultivar ‘Aegina’ extracted with two different techniques (UAE, Soxhlet) were tentatively identified and quantified as percentage of the total volatiles by HS-SPME combined with GC-MS technique. The results showed that there were differences between the two types of extraction. The major compounds were α-pinene, octane, decane for UAE and decane, nonanal, (*E*)-2-decenal for Soxhlet. When the pistachio oil had undergone a more intense thermal treatment (Soxhlet), the content of aldehydes and hydrocarbons increased, but the levels of terpenes decreased. The literature showed similar groups of volatile compounds as compared to those found in the present study.

## Author contribution statement

Lydia Valasi: Conceived and designed the experiments; Performed the experiments; Analyzed and interpreted the data; Contributed reagents, materials, analysis tools or data; Wrote the paper.

Evangelia C. Zafeiri, Ioanna Thanou: Performed the experiments; Analyzed and interpreted the data.

Christos Pappas: Conceived and designed the experiments; Contributed reagents, materials, analysis tools or data.

## Data availability statement

Data included in article/supplementary material/referenced in article.

## Funding

This research did not receive any specific grant from funding agencies in the public, commercial, or not-for-profit sectors.

## Abbreviations

HS-SPME: headspace solid-phase microextraction
GC-MS: gas chromatography-mass spectrometry
UAE: ultrasound assisted extraction
PKO: pistachio kernel oil
KRI: Kovats retention index/indices
PTFE: polytetrafluoroethylene
DVB: divinylbenzene
CAR: carboxen
PDMS: polydimethylsiloxane
